# Validity and Reliability of the Turkish Version of the Modified Breast Cancer Worry Scale

**Published:** 2018-11

**Authors:** Sermin TIMUR TAŞHAN, Tuba UÇAR, Yeşim AKSOY DERYA, Gülçin NACAR, Behice ERCI

**Affiliations:** 1. Dept. of Women Health and Diseases Nursing, Faculty of Nursing, Inonu University, Malatya, Turkey; 2. Dept. of Midwifery, Faculty of Health Sciences, Inonu University, Malatya, Turkey

**Keywords:** Breast cancer worry scale, Cancer worry, Turkish version, Validity, Reliability

## Abstract

**Background:**

There are many assessment instruments used for cancer worry. Many women worry about breast cancer, but In Turkey, there is no scale that assesses the worry about developing breast cancer. The aim of this study was to investigate the validity and reliability of the Turkish Breast Cancer Worry Scale (BCWS). This scale was created as a modified version of the Cancer Worry Scale.

**Methods:**

The study was conducted in a Family Health Center (FHC) located in eastern Turkey using a methodological design. The study sample consisted of 610 healthy women who referred to a FHC for any reason. The data were collected using the Participant Information Form and BCWS with a face-to-face interview conducted between Jun 2015 and Jan 2016. Construct validity of the scales was tested via factor analysis, Cronbach’s alpha coefficient, item-total score correlation coefficient, and test-retest correlations were calculated to check for reliability.

**Results:**

The factor load values of BCWS were found to be between 0.45 and 0.79. The total Cronbach’s alpha coefficient of the scale was 0.78, and the total score correlations of items ranged between 0.32 and 0.64. The test-retest reliability coefficient of the scale was 0.81 (*P*=0.001).

**Conclusion:**

The Turkish version of the BCWS is a valid and reliable tool for assessing the effect of breast cancer worry on daily activities and mental condition.

## Introduction

The incidence of breast cancer, which is the most common cancer type observed in women, is increasing rapidly in the world and in Turkey and about 1 million new cases are reported each year (1). Many women worry about contracting breast cancer (2). The keyword that directs individuals to early diagnosis and treatment and enables them to employ a health-promoting lifestyle is the worry about getting cancer (3). This emotion causes behavioral changes and can have a motivating influence on adopting health-protective behaviors (4). Recent studies have emphasized the effect of worry on the prevention and early diagnosis of cancer (2–7). The worry of individuals has an effect on adopting health-protective behaviors against breast cancer (2–6).

Since the worry is subjective, the way individuals perceive this emotion is also of great importance. There are many assessment instruments in the literature used for cancer worry. The Cancer Worry Scale (CWS), an assessment tool developed (8, 9), was proved to be valid and reliable for cancer patients in general. Lerman modified the scale from breast cancer to general cancer and increased the number of questions to six. In Turkey, there is no scale that assesses the worry about developing breast cancer; therefore, qualified assessment tools are required to measure the breast cancer worry. It is important that the cultural adaptation of a scale possesses a cultural fit, in other words, its preparation for use in different cultural contexts.

This study aimed to determine the validity and reliability of the Turkish version of Breast Cancer Worry Scale (BCWS), which is a modification of CWS.

## Materials and Methods

### Design and participants

This study was conducted in a Family Health Center (FHC) located in an eastern city of Turkey using a methodological design. The study sample included 610 healthy women who referred to a FHC for any reason, who complied with the inclusion criteria of the study and who agreed to participate in the study. The inclusion criteria of the study was not having been diagnosed with breast cancer previously.

In validity and reliability studies, it is important for the sample size to be sufficient so that correlations can be examined reliably. As a general rule, the sample size should be five times the number of items on the scale (10). Moreover, studying larger samples helps to obtain more reliable results and suggested that the subject-to-variable ratio that is considered for sample should be at least 10:1 (11). Including 60 women in the present study was found to be acceptable because the item number of BCWS was six. However, the number of samples was increased to 610 to assist the confirmatory factor analysis to become more reliable.

### Instruments

The data were collected by the researchers between Jun 2015 and Jan 2016 using the Participant Information Form and the Breast Cancer Worry Scale (BCWS) in face-to-face interviews.

### Participant Information Form

This form consisted of questions about the sociodemographic characteristics (age, occupation, educational level, and income level) of the women who participated in the study.

### Breast Cancer Worry Scale (BCWS)

The first version of BCWS that included 3 items was developed. The BCWS measures the effect of breast cancer worry on daily activities and mental condition and its Cronbach’s alpha reliability coefficient is 0.86 (9). The BCWS was modified and generalized it to all cancer types by increasing the number of questions to six and it was called the Cancer Worry Scale (CWS) in 1994. A 5-point Likert-type scale was used in the CWS and it included the response options for each question: never=0, rarely=1, sometimes=2, often=3 and 4=always. The score given for each question is taken into consideration. Thus, the lowest possible score obtained from the scale is 0, whereas the highest score is 24. A total score lower than 12 indicates a low level of cancer worry, whereas a scale that is 12 or higher indicates a high level of cancer worry. The CWS includes the following five questions: “How often have you thought about your chance of getting cancer?”, “Have these thoughts affected your mood?”, “Have these thoughts interfered with your ability to do daily activities?”, “How concerned are you about the possibility of getting cancer one day?”, “How often do you worry about developing cancer?” and “How much of a problem is this worry?” (8, 9).

The item number, the rating and the assessment of the Turkish version of the BCWS, which was modified from Lerman’s CWS, were identical to those of the CWS except that the word *cancer* used in the CWS was changed to *breast cancer* in the Turkish version of BCWS. The short and reliable CWS is one of the first assessment tools that measure cancer worry (8).

### Process of cultural adaptation

The cultural adaptation of BCWS was conducted according to the stages described by (10). These stages included: 1) translation of the scale from English into Turkish by two independent linguists; 2) preparation of the Turkish text that represented each item in the best possible way and review of two translations; 3) back-translation of the scale into English by two independent linguists; 4) presenting the Turkish form of the scale for academic members’ opinions to determine its cultural suitability; 5) conducting a preliminary test using the scale arranged according to the academic members’ recommendations; 6) analyzing the psycho-metric properties of the finalized Turkish version of BCWS (validity and reliability study).

The BCWS was translated from English into Turkish by two linguists. Afterward, five academic members from gynecology and obstetrics nursing, midwifery and community health nursing departments gathered and reviewed both translations. After the review process, the first Turkish draft of the BCWS that represented each item in the best possible way was prepared. Then, the Turkish draft was back-translated into English. Translations were done by two independent linguists. The back-translation of the scale was observed to be consistent with the original BCWS. The linguists who translated the BCWS from English into Turkish and translated it back from Turkish into English were informed about the study. These translators were academic members in the Department of English Language and Literature at a university and they were teaching courses in the field of health so they had known about the field.

Expert opinions were received from 10 academic members from the fields of Gynecology and obstetrics nursing and community health nursing to determine the cultural suitability of BCWS. The scale was sent to them via email. The expert academic members were asked to score each question of the scale from 1 to 4 to assess the cultural suitability of the scale items. This scoring corresponded to the following responses, respectively: “not suitable”, “slightly suitable, but the item should be made more suitable”, “quite suitable but minor changes should be done” and “very suitable” (12). The concordance level of expert opinions was evaluated using a nonparametric test, Kendall’s W analysis (13, 14). There was no statistical difference between the scores given by the experts (Kendall W=0.08; *P*>0.05) so the experts were consistent with their assignment of scores. In this stage, a pre-final form of BCWS was prepared in accordance with the expert comments about the scale items.

The preliminary test of this scale was performed on ten women. It took approximately five minutes for each participant to answer the questions using the scale. The results were not included in the sample. No question was misunderstood in the preliminary test of the scale. Thus, the Turkish version of BCWS was put into its final form.

### Psychometric testing of the BCWS Validity

To determine the construct validity of the scale, factor analysis was conducted. Prior to this analysis, the sampling adequacy of the study was determined using the Kaiser-Meyer-Olkin (KMO) analysis and the sample test size was determined using Bartlett’s Test of Sphericity. The KMO was found to be higher than 0.60. The result of Bartlett’s Test of Sphericity was found to be statistical significant, which indicated that the sample size was adequate (14, 15).

The Principal Component Analysis, one of the most commonly used factor analysis statistical techniques, was used to examine the factor structure of the BCWS. Items with a factor load lower than 0.30 in the factor analysis should be removed from the scale (8, 16).

### Reliability

The Cronbach’s alpha technique is recommended for the examination of Likert-type scales. The reliability coefficient should be as close to 1 as possible for an assessment instrument to be regarded as adequate (12, 13). If the Cronbach’s alpha coefficient is lower than 0.40, the assessment instrument is not reliable. If it is between 0.40 and 0.59, the instrument’s reliability is low. If it is between 0.60 and 0.79, the instrument is quite reliable, and if it is between 0.80 and 100, the instrument is considered to be extremely reliable (12, 14).

To examine the correlation between the scores obtained from the test items of the BCWS and its total score, item-total score correlation coefficients were determined. The acceptable coefficient for item selection is greater than 0.20 (13, 14).

Three weeks later, the scale was administered again to 30 women to conduct the test-retest analysis of the BCWS. The time invariance of the scale was assessed using test-retest correlation and the *t*-test.

### Data analysis

Study data were analyzed using SPSS 16.0 for Windows software (Chicago, IL, USA). In the present study, this software was used for the analysis of the psychometric properties of BCWS as well as for the analysis of the descriptive statistics (number, percentage, mean, and standard deviation) utilized for the introductory characteristics of the participants. The significance level was set to be 0.05.

### Ethical approval

Written permission was obtained from Caryn Lerman via e-mail to conduct the adaptation study of the Breast Cancer Worry Scale into Turkish and to determine its validity and reliability. Ethical approval was obtained from the Ethics Committee of University Institute of Medical Sciences (2014/44). A written approval was obtained from The Public Health Agency of Turkey and from the Family Health Center to conduct this study. An informed consent form was read to all participants and their verbal and written consents were received in the data collection process. The data obtained would be published for scientific aims without using the names of the participants.

## Results

### Descriptive characteristics of the sample

The sociodemographic characteristics of the women and their behaviors related to breast cancer early diagnosis methods are shown in Table 1. The mean age of the women was 39.18±1.19 yr. Of them, 75.5% were housewives, 50.3% had low levels of income and 49.8% were high school or university graduates (Table 1).

**Table 1:** Descriptive characteristics of the women (n=610)

***Characteristics***	***X ± SD***	***Min-Max***
Age (yr)	39.18±1.19	(18–68)
	**n**	**%**
Occupation		
Unemployed	460	75.5
Employed	150	24.5
Family income		
Low	307	50.3
Medium/ High	303	49.4
Educational level		
No education or literate	54	8.8
Primary school graduate	253	41.4
High school or university graduate	303	49.8

### Validity

In the present study, the results of the KMO analysis and of Bartlett’s Test of Sphericity were found to be 0.756 and 1194.6, respectively. Both test results were found to be significant at the level of *P*=0.001. The sample size is adequate and suitable for factor analysis.

As a result of this factor analysis conducted to determine the validity of the Breast Cancer Worry Scale, the factor load values were found to be between 0.45 and 0.79 and to explain 70.02% of the total variance (Table 2). Thus, one-dimensional Breast Cancer Worry Scale consisting of 6 items was obtained.

**Table 2:** Factor loadings and item-total correlations of the breast cancer worry scale (n= 610)

***Scale item***	***Mean***	***SD***	***Factor Loading***	***Corrected Item-total Correlations***
1. How often have you thought about your chances of getting *breast* cancer?	0.91	1.05	0.457	0.321
2. Have these thoughts affected your mood?	1.45	1.31	0.793	0.645
3. Have these thoughts interfered with your ability to do daily activities?	1.07	1.32	0.741	0.573
4. How concerned are you about the possibility of getting *breast* cancer one day?	0.78	1.04	0.574	0.435
5. How often do you worry about developing *breast* cancer?	1.64	1.31	0.788	0.646
6. How much of a problem is this worry?	1.74	1.45	0.767	0.604

Variance = 70.02%

Cronbach’s alpha= 0.78

### Reliability

The Cronbach’s alpha reliability analysis was conducted to assess the internal consistency of the 6-item BCWS and it showed that the reliability coefficient of the scale was 0.78 (Table 2). The correlation of the scale items ranged between r=0.32 and 0.64, and the correlation between each item and the total score was statistically significant (*P*=0.001) (Table 2).

The assessment of the results of two measurements of the scale was administered at a 3-week interval and it showed that there was no statistically significant difference between the scale mean scores obtained from the first (pretest) and second (post-test) applications (*t*=−1.123, *P*=0.271). Moreover, the correlation value (r=0.81, *P*=0.001) between the first and second test scores of the BCWS was found to indicate a strong, positive and statistically significant correlation.

## Discussion

In Turkey, there is a requirement for qualified assessment tools used to measure the breast cancer worry. The most important features expected from a measurement tool are validity and reliability. Thus, this study aimed to determine the validity and reliability of the Turkish version of the Breast Cancer Worry Scale modifying the Cancer Worry Scale as the Breast Cancer Worry Scale and examined the psychometric properties of the Turkish version of the scale. The BCWS was found to be both valid and reliable in general.

### Validity

To determine the BCWS construct validity, a scale’s ability to measure the relevant concept and the entire conceptual structure, a factor analysis was conducted. In this study, the factor load values of BCWS were found to be between 0.45 and 0.79. No item was removed from the scale because there was no item the factor load of which was lower than 0.30. A study was conducted to determine the validity and reliability of the Spanish version of the Breast Cancer Worry Scale and they found the factor load values of the scale to range between 0.63 and 0.82 (17). In a validity and reliability study conducted in Germany, two items were added to the Breast Cancer Worry Scale and the factor load values of the 8-item scale were found to range between 0.42 and 0.87 (8). These results show similarity with the results obtained from the present study.

This study found that the BCWS is one-dimensional as it is in the original scale and explains 70% of the total variance. The construct validity of the scale was ensured considering that 30% and higher is regarded as a criterion for the ratio of variance explained in scale development and adaptation studies (16). Similarly, the scale was found to explain 53.07% of the total variance in its Spanish version (17) and 55.2% of the total variance in its German version (8). The BCWS was used in a study they conducted in England to examine the attitudes of women toward breast cancer tests (18). In this study, the scale was found to explain 61% of the total variance.

### Reliability

To assess the reliability of the scale, the Cronbach’s alpha reliability coefficient, item-total score correlation, and test-retest analysis were conducted (13).

The Cronbach’s alpha reliability coefficient should be as close to 1 as possible for an assessment instrument to be regarded as adequate (13, 14, 19) and a value of 0.7 or above was considered to indicate good reliability (20). In the present study, the Cronbach’s alpha reliability coefficient of the BCWS was found to be 0.78, which shows that the scale is highly reliable. The Cronbach’s alpha reliability coefficient was found to be 0.83 in the Spanish version of the scale (17) and 0.87 in its German version (8). The Cronbach’s alpha reliability coefficient ranged between 0.77 and 0.89 in many studies that used the scale (21–23).

Item–total score correlation coefficients explain the correlation between the scores obtained from the test items and its total score. A positive and high item–total score correlation indicates that the items have drawn out similar responses and the internal consistency of the test is high. Item–total score correlation is calculated using the Pearson’s correlation coefficient in tests where Likert-type rating scales are used. When the correlation obtained for each item is high, the relationship of that item with the measured theoretical structure is also high; in other words, the item is effective in and adequate for measuring the intended behavior. The acceptable coefficient for item selection is greater than 0.20 (13, 14, 19). The reliability coefficients of the BCWS were found to range between r=0.32 and 0.64, and the correlation between each item and the total score was observed to be statistically significant (*P*=0.001).

The scale was administered to 30 women three weeks after the first application to determine the time invariance of the BCWS. The value of the correlation between the first and the second tests was high and there was a significant relationship between them (r=0.81, *P*=0.001), which shows that the scale provided consistent results and it is time invariant. Similarly, the correlation coefficient was found to be 0.77 in the Spanish version of the scale (17). A study conducted in South East Scotland using the scale found a correlation coefficient of 0.74 (22).

## Conclusion

The Turkish version of the BCWS is a valid and reliable tool to assess the effect of breast cancer worry on daily activities and mental condition. This scale can be used in studies conducted to assess the breast cancer worry. Revealing the suitability of the scale for Turkish culture produced evidence that it can be used in similar cultures. In this respect, BCWS is important for international literature. In addition, studies should be carried out to test the validity and reliability of the scale in different samples.

## Ethical considerations

Ethical issues (Including plagiarism, informed consent, misconduct, data fabrication and/or falsification, double publication and/or submission, redundancy, etc.) have been completely observed by the authors.
